# Inter‐seasonality of influenza in Australia

**DOI:** 10.1111/irv.12642

**Published:** 2019-03-30

**Authors:** Aye M. Moa, Dillon C. Adam, C. Raina MacIntyre

**Affiliations:** ^1^ Faculty of Medicine, The Kirby Institute University of New South Wales Sydney New South Wales Australia; ^2^ College of Public Service and Community Solutions Arizona State University Tempe Arizona

**Keywords:** Australia, influenza, inter‐seasonality

## Abstract

**Background:**

It appears inter‐seasonal influenza notifications have been increasing in summer months in Australia. This study aims to determine changes in inter‐seasonal influenza activity in Australia over time.

**Methods:**

Routine influenza surveillance data and hospitalisations data were analysed to study the epidemiology of inter‐seasonal influenza and to examine the impact of inter‐seasonal influenza on morbidity in Australia at a national level. To adjust for changes in testing over time, we calculated a ratio of summer‐to‐winter notifications for each year in the study. A *P*‐value of <0.05 was used for statistical significance.

**Results:**

Nationally, 18 933 notifications were reported during summer months from December to February 2005‐2016. There have been increasing summer notifications over time, which corresponded to similarly increased notifications in winter. A significant upward trend was observed for rate of notification during summer period over these years, *P* < 0.01. However, the ratio of summer‐to‐winter notifications demonstrated that while notifications have increased, the ratio has not increased markedly over the years and did not show a significant trend. No seasonal trend in rates of hospitalisation for influenza and pneumonia, respiratory and circulatory diagnosis was observed over the studied years.

**Conclusion:**

This study provides a clearer understanding of the epidemiology and burden of inter‐seasonal influenza and trends over time in Australia. The ratio of summer‐to‐winter notifications remains relatively constant and is supported by reasonably constant hospitalisation rates over the years.

## 1 BACKGROUND

Influenza is an epidemic infection which affects millions of people around the world annually. Globally each year, the incidence of human influenza rises and falls in temperate regions during winter.^1^ This is known as seasonality and is largely believed to be driven by favourable climate conditions for viral survival and transmission,^2^ and changes in population behaviours such as crowding.^3^ In tropical and subtropical regions, however, this distinct seasonal pattern is less clearly defined^4^ with cases occurring year round, particularly in tropical East and South‐East Asia.^5^ Inter‐seasonal notifications of influenza, in the summer months, were rare a decade ago.^6^ However, notifications of influenza appear to have increased in summer and outside of the usual influenza season in Australia.^7^ Australia is a large continental land, which spans many climatic zones, from tropical and subtropical, to temperate and cool temperate. In temperate and cool temperate Australia, the seasonal rise in influenza incidence typically begins around May and falls by October.^8^ Cases that occur outside this period are characterised as inter‐seasonal migrant viruses—imported from the opposing hemisphere or the tropics.^9^ Travel has increased exponentially over the last two decades, with about 766 600 short‐term visitor arrivals to Australia during July 2018, an increase of approximately 43% when compared with records within the last two decades.^10^ Inter‐seasonal cases are often sporadic and thought to be unviable for ongoing transmission, and hence, their lineages become extinct.^11^ However, a recent study has shown that in some instances, lineages of inter‐seasonal influenza in Australia have continued to circulate into the typical winter season.^7^ Furthermore, in some cases, seasonal lineages of influenza B demonstrated persistent transmission across the inter‐seasonal period, effectively bridging one season to another. If inter‐seasonal lineages can be shown to persist into and across typical seasonal periods, this gives rise to questions of seasonal onset. One recent study has investigated the onset of seasonal influenza across each climatic zone in Australia. They showed the onset of epidemics varies season to season; however, there is remarkable synchronisation of epidemic onset across Australia, with a winter peak typically in August, despite the significant distance involved and variation in climate.^12^ There have also been reported increased cases of summer influenza or out‐of‐season epidemic in other countries.^13^ We aimed to determine whether inter‐seasonal influenza activity has been increasing in Australia over time.

## 2 METHODS

Laboratory‐confirmed influenza notifications and notification rates per 100 000 population were extracted from the National Notifiable Diseases Surveillance System (NNDSS), Department of Health, Australian Government.^14^ Influenza has been a notifiable disease in Australia since 2007, and all states and territories notified cases to state health department and then to the Department of Health and collated data nationally. A national surveillance case definition for influenza is described below. Only confirmed cases are notified and a confirmed case requires definitive laboratory evidence^15^:

(a) Isolation of influenza virus by culture from appropriate respiratory tract specimen, or (b) detection of influenza virus by nucleic acid testing from appropriate respiratory tract specimen, or (c) laboratory detection of influenza virus antigen from appropriate respiratory tract specimen, or (d) IgG seroconversion or a significant increase in antibody level or a fourfold or greater rise in titre to influenza virus and or (e) single high titre by complement fixation test or haemagglutination inhibition assay to influenza virus. The threshold for a high titre could vary over time and between states and territories.

Every year, influenza seasonal activity occurs between May and October in Australia, with a typical peak in August.^16^ Inter‐seasonality was defined as influenza activity occurring in the period from December to February and was compared to influenza activity during the winter months (June‐August). The NNDSS data, which are validated and confirmed as influenza, were analysed to examine an association between inter‐seasonality of influenza and the impact on morbidity. The 2009 pandemic year was excluded from the analysis, which was restricted to seasonal influenza in the study.

Routine data sources including national hospitalisations data from the Department of Health, AIHW,^17^ and population data from the Australian Bureau of Statistics (ABS), Australian Government were also used in the study.^18^ Weekly hospitalisations data were extracted for three disease categories, using the clinical diagnosis coding ICD‐10‐AM with regard to the principal diagnosis of influenza and pneumonia (J09–J18), all respiratory (J00–J99) and circulatory disease (I00–I99) for all age group. Data were then aggregated and analysed as monthly hospitalisations in the study. We used monthly rate of hospitalisation per 100 000 population in each diagnosis category for summer period from December to February, and for winter from July to August, between 2005 and 2016. We tested whether there was a seasonal trend in the rate of hospitalisation during summer.

Data from both sources (NNDSS and AIHW) were then analysed to study the epidemiology of inter‐seasonal influenza from 2005 or 2005/2006 to 2016.

### 2.1 Analysis methods

A descriptive analysis was conducted using the notification and hospitalisation data by year. One of the reasons for increased summer notifications is increased testing for influenza over time. To adjust for changes in testing over time, we calculated an adjusted ratio (ratio of summer‐to‐winter notifications) for each year assuming that a real increase in summer cases may be reflected in an increased ratio over time. We tested for trend using the seasonal Mann‐Kendall test to detect trends over time during the studied years using xlstat software.^19^ IBM spss Statistics, Version 22 was used,^20^ and a *P*‐value of <0.05 was applied for the statistical significance.

## 3 RESULTS

A total of 18 933 notifications were reported nationally in summer months from December to February during the study period, 2005‐2016. A minimum of 174 in 2006 and a maximum of 4071 notifications reported in 2014, with a mean value of 1893. Table 1 shows the number of laboratory‐confirmed influenza notifications and notification rate per 100 000 population during summer period (December‐February) and winter months (June‐August), and an adjusted ratio from 2005 to 2015/2016 in Australia. Over the study period, the number of notifications and notification rate varied from year to year; however, the number increased in later years especially after the pandemic year, 2009 (Table 1). We tested notifications for trend over time during the summer months using the Seasonal Mann‐Kendall test, and the results showed that there was a trend to increased summer notifications, which was statistically significant (*P* < 0.01). Similarly, a significant trend was also observed for notification rate during the summer period over the years, *P* < 0.01. Table 1 indicates that while the notifications are increased, the ratio of summer‐to‐winter notifications or the adjusted ratio has not increased markedly. Besides, testing of the adjusted ratio for seasonal Mann‐Kendall test over the years did not show a significant trend, *P* = 0.13.

**Table 1:** Laboratory‐confirmed influenza notifications and notification rate per 100 000 population, and adjusted ratio during summer and winter months, Australia, 2005/06‐2015/2016 excluding the 2009 pandemic

Year	Summer^a^		Year	Winter^b^		Adjusted ratio
	Notifications	Notification rate		Notifications	Notification rate	
2005/2006	174	0.9	2005	2889	14.2	0.06
2006/2007	291	1.4	2006	1873	9.1	0.16
2007/2008	392	1.8	2007	7990	38.4	0.05
2008/2009	496	2.3	2008	3559	16.7	0.14
2010/2011	2682	12.1	2010	3496	15.9	0.77
2011/2012	1106	5.0	2011	15 929	71.3	0.07
2012/2013	2105	9.2	2012	33 779	148.7	0.06
2013/2014	4071	17.4	2013	11 694	50.6	0.35
2014/2015	3723	15.6	2014	38 924	165.8	0.10
2015/2016	3928	16.2	2015	57 523	241.6	0.07

Adjusted ratio = ratio of summer‐to‐winter notifications.

a Summer months (December‐February).

b Winter months (June‐August).

**Figure 1:**
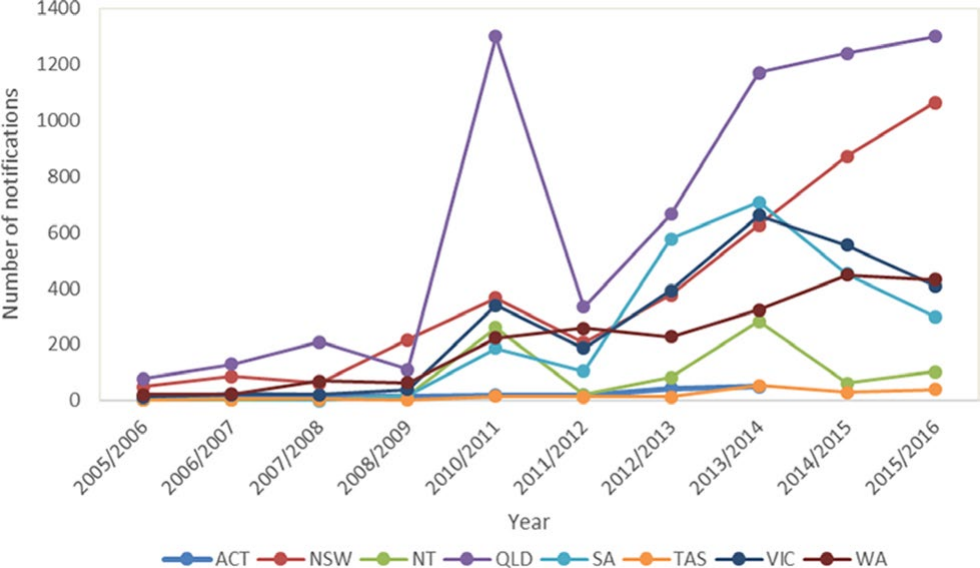
Laboratory‐confirmed influenza notifications during summer^†^ by states and territories, and by year, Australia, 2005/2006‐2015/2016 excluding the 2009 pandemic. Footnote: data not available for ACT in years 2014/2015 and 2015/2016; ^†^Summer period (December‐February)

We analysed summer notifications across all states and territories in Australia over the study period. Figure 1 presents the variation in the number of notifications among states and territories over the studied years during the summer months (December‐February). The data showed that there was an increase in notified cases after 2009. There was a statistically significant difference in mean notifications by the states before and after the pandemic year, 2009, *P* = 0.02. Across all states and territories, Queensland had the highest number of notified cases (n = 6534) over the period in the study (Figure 1). Although notifications were increased in the later years, the adjusted ratio for seasonal Mann‐Kendall test did not show a significant trend for QLD, *P* = 0.76.

Table 2 shows rate of laboratory‐confirmed influenza notification per 100 000 population across Australian states and territories during summer. Unusually, high notification rates were seen in the state of Northern Territory during the summer months of 2010/2011 and 2013/2014 (Table 2). Nationally, an average annual summer notification rate was 8.2 per 100 000 population. Among the states and territories, Northern Territory had the highest summer notification rate followed by Queensland and South Australia with an average notification rate of 36.1, 14.3 and 13.4 per 100 000 population per year, respectively. A statistically significant trend in summer notification rates was also observed in all states and territories over the years (Table 2).

**Table 2:** Summer notification rate by states and territories, by year, Australia, 2005/2006‐2015/2016 excluding the 2009 pandemic

Year	States and territories								Australia
	ACT	NSW	NT	QLD	SA	TAS	VIC	WA	
2005/2006	1.8	0.7	3.8	2	0.1	0.2	0.2	1	0.9
2006/2007	3.6	1.3	2.3	3.3	0.4	0.6	0.5	1.1	1.4
2007/2008	4.7	1	10	4.9	0	0.8	0.4	3	1.8
2008/2009	4	3.4	8	2.5	1	0.2	0.7	2.9	2.3
2010/2011	4.4	5.1	114.8	28.6	10.7	2.6	6.3	9.3	12.1
2011/2012	4.6	2.9	6.9	7.5	6.5	2.6	3.1	9.7	5.0
2012/2013	9.7	4.6	27.7	13.3	28.3	2	6.1	8.5	9.2
2013/2014	12.9	9.2	122.9	26.3	42.5	10.7	11.9	13.8	17.4
2014/2015	19.1	11.6	24.6	26	26.6	5.5	9.1	17.7	15.6
2015/2016	12.9	14.8	40	28.9	18.3	7.3	7	17.7	16.2
Annual average rate	7.8	5.5	36.1	14.3	13.4	3.3	4.5	8.5	8.2
Trend test^a^ (*P*‐value)	0.04	<0.01	0.04	0.04	<0.01	0.01	<0.01	<0.01	<0.01

Summer months (December‐February).

a Mann‐Kendall seasonal trend test; Rate per 100 000 population.

A descriptive summary of hospitalisation rates across three diagnosis categories over the studied years is shown in Table 3. A mean hospitalisation rate of 64.2/100 000 population in influenza and pneumonia to a mean rate of 509.9/100 000 population in circulatory hospitalisations per year were observed over the years. There was no significant trend in increased summer rates of hospitalisations for all diagnosis categories during the studied years (Table 3).

**Table 3:** A descriptive summary of rate of hospitalisation by disease category, Australia, excluding the 2009 pandemic

	Summer months (December‐February)	Minimum	Maximum	Mean	Trend test^a^ (P‐value)
Influenza and pneumonia^b^ hospitalisation rate	2005/2006‐2013/2014	55.2	76.9	64.2	0.56
Respiratory^c^ hospitalisation rate	2005/2006‐2013/2014	314.7	377.1	344.4	0.08
Circulatory^d^ hospitalisation rate	2005/2006‐2013/2014	435.8	536.8	509.9	0.56

Rate per 100 000 population.

a Mann‐Kendall seasonal trend test.

b ICD‐10‐AM code: J09–J18.

c ICD‐10‐AM code: J00–J99.

d ICD‐10‐AM code: I00–I99.

**Figure 2:**
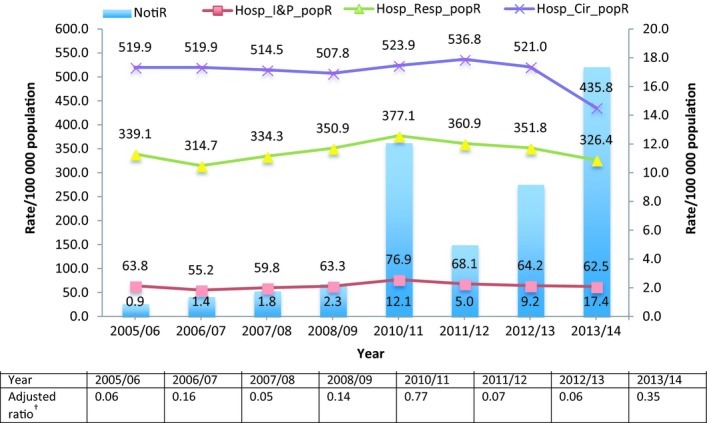
Influenza and pneumonia (IP), respiratory and circulatory hospitalisation rates, notification rate and an adjusted ratio during summer months, by year, Australia, 2005/2006‐2013/2014 excluding the 2009 pandemic. Hosp_Cir_popR, Circulatory hospitalisation rate (ICD‐10‐AM code: I00–I99); Hosp_I&P_popR, Influenza and pneumonia hospitalisation rate (ICD‐10‐AM code: J09–J18); Hosp_Resp_popR, Respiratory hospitalisation rate (ICD‐10‐AM code: J00–J99); NotiR, Notification rate. Footnote: ^†^Adjusted ratio = ratio of summer‐to‐winter notifications

Figure 2 also demonstrates hospitalisation rates per 100 000 population during summer (December‐February), with principle diagnosis category of influenza and pneumonia, respiratory and circulatory admissions, and the notification rate and the adjusted notification ratio by year in Australia, and shows that these summer hospitalisation rates have not increased over the years.

## 4 DISCUSSION

This study provides a clearer understanding of the epidemiology and burden of inter‐seasonal influenza in Australia and trends over time. While summer influenza notifications and notification rates have increased significantly over time, especially since the 2009 pandemic, there has been a similar increase in winter notifications over the years. The ratio of summer‐to‐winter notifications remains relatively constant over the studied years, suggesting that the increase in notifications reflects an increase in testing over time. This is supported by relatively constant summer hospitalisation rates for influenza and pneumonia over the study periods.

The state‐based data showed varied notification rates as well as significant increasing trend in summer notification rates across the states over time. Another Australian study found that there was marked increase in influenza notifications in Australia during summer period of 2010‐2011; however, authors reported that those high notifications were simply due to variation in virus circulation during summer and no association or impact had been reported with regard to increased cases.^21^ Similar to our study, evidence of increased notifications was reported in Australia after the pandemic.^21^, ^22^ Testing for influenza may have increased after the pandemic due to increased awareness of the disease and the disease impact in the society. In addition, availability and use of rapid point of care tests for influenza have also increased since 2009 after the provision of public funding for rapid testing by the government.^22^

Some countries experience more than one peak of influenza activity per year. Countries close to the equator and the tropical countries demonstrate a persistence of year‐round influenza activity or multiple seasonal peaks of influenza each year.^23^ For example, tropical and subtropical countries such as Thailand and Hong Kong have two seasonal peaks of influenza per year.^24^ In temperate regions, outbreaks of influenza A and B are also reported during summer.^13^, ^25^, ^26^, ^27^ Australia typically has a single winter peak, and summer influenza outbreaks are uncommon. Globally, there are reports of summer influenza or out‐of‐season outbreaks in institutions, as well as among travellers on cruise ships.^26^, ^28^, ^29^ International travel is a source of importation of influenza during the Australian summer, and the public health implications of inter‐seasonal influenza activity need to be better understood to inform use of influenza vaccine and other preventive measures for influenza control and prevention.

There are some limitations in our study. Our study used laboratory‐confirmed cases notified nationally; however, influenza notifications may not reflect the true incidence of influenza infections in the population. Moreover, diagnostic practices by general practitioners such as the use of rapid point of care tests, test types used by laboratories and by testing practices by regional health jurisdiction may have changed over time and vary across the country. The increase in notifications received by NNDSS nationally over time must therefore be interpreted with caution. In addition, our study did not include any genetic sequence data or phylogenetic analysis of influenza virus circulating during summer periods, which could add further information. For example, recent studies in Australia have provided evidence of ongoing transmission of summer month influenza into the traditional winter season and vice versa.^7^ Furthermore, influenza B circulation specifically has been shown to occasionally persistent between yearly winter seasons, traversing the intermediate summer season entirely.^7^ Phylodynamic modelling could be used to confirm whether the incidence of summer month influenza has remained steady over time as indicated by stable viral population size^32^ or viral sampling proportions^33^ across seasons. Considering the aforementioned persistence, uncovering the true summer incidence of influenza B viruses in Australia particularly warrants further investigation.

To conclude, we found increasing summer notifications over time, which corresponded to similar increased notifications in winter, without changes in influenza hospitalisations over the same period. This supports the conclusion that the apparent increase in incidence of influenza during summer is a result of increased testing over time. These results should be interpreted carefully in conjunction with other surveillance systems to understand the overall epidemiology of influenza infection in the country.
